# Use of seated positioning device (Smoltap^®^) for ultrasound-guided infant lumbar punctures

**DOI:** 10.1007/s00247-025-06291-6

**Published:** 2025-06-09

**Authors:** Shimwoo Lee, Joseph Miller

**Affiliations:** Department of Interventional Radiology, Children’s Hospital of Los Angeles, 4650 Sunset Blvd, Los Angeles, CA 90027 United States

**Keywords:** Lumbar puncture, Infant, Seated position, Device, Ultrasound guidance

## Abstract

**Background:**

Infant lumbar punctures (LPs) frequently fail at bedside and prompt repeat attempts with image guidance. Conventionally, image-guided LPs are performed with ultrasound or fluoroscopy while infants are in lateral flexed position. The procedure requires infants to be either sedated or held manually to maintain stable positioning. A new commercially available positioning device (Smoltap^®^) provides an alternative method to secure infants in sitting position without needing to administer sedation.

**Objective:**

To evaluate the effectiveness and safety of an infant positioning device during image-guided LPs as an alternative to the conventional LP technique.

**Materials and methods:**

We conducted a retrospective analysis of image-guided LPs from May 2022 to April 2025, approximately 1.5 years before and after the introduction of an infant positioning device in October 2023 at our institution. The device was used for awake infants stable on room air and with head-to-toe length < 57 cm, per instructions for use. The infants were secured in the device, and LPs were performed with ultrasound guidance. Patient demographics and procedural outcomes of LPs performed with and without the device were compared.

**Results:**

We analyzed 42 LPs performed with the device (“device” group) and 37 LPs performed without (“no device” group). The two groups had similar patient characteristics and rates of prior failed bedside LPs (95% and 86%, respectively, *P* = 0.17). The success rates of obtaining adequate CSF for microbial culture were comparable between the groups (93% and 84%, *P* = 0.21). There were no complications in either group. The average procedure duration was also similar (14 min vs 16 min, *P* = 0.65). There was no statistically significant difference in the proportions of traumatic taps when defined as CSF containing ≥ 10,000 erythrocytes/µL (26% and 16%, *P* = 0.42). When defined as ≥ 500 erythrocytes/µL, the rates of traumatic taps were significantly different (72% and 42%, *P* = 0.01). Subgroup analysis of the “no device” group suggested that this difference could be attributed to inclusion of sedated patients in the “no device” group. In the “device” group, no patients received sedation.

**Conclusion:**

Performing image-guided infant LPs with a positioning device is a feasible and safe alternative to the conventional technique with the benefit of not needing to sedate or manually hold infants.

**Graphical Abstract:**

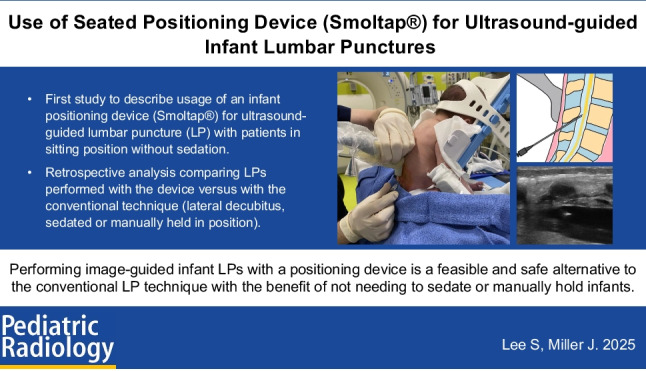

## Introduction

Lumbar punctures (LPs) are frequently performed in infants to evaluate for bacterial meningitis, which is associated with significant mortality and morbidity if untreated. However, approximately 40% of LPs in infants are unsuccessful [1, 2]. Failed LPs at bedside often prompt consultation to interventional radiology (IR) for image-guided LPs. The standard LP technique in most IR practices is to use either ultrasound or fluoroscopic guidance while the infant is in a lateral flexed position. Although the procedure technique is relatively straightforward, the resources and labor required to support the procedure is not insignificant. Either the infant needs to be sedated by an anesthesiologist or held manually by a trained staff in order to maintain the lateral flexed position for the entire duration of the procedure.

Several studies have demonstrated the benefits of the sitting position for infant LPs. The largest randomized controlled trial to investigate infant LPs with > 1,000 patients (NeoCLEAR) showed that the sitting position was associated with higher rates of successful first LPs and fewer rates of desaturation during procedures [3]. A smaller study involving 132 infants also found greater likelihood of obtaining CSF on the first LP attempt with the sitting position compared to lateral, although the overall success rate was similar [4]. An older study evaluating hypoxemia events in infants during LPs showed lower mean transcutaneous pO2 for the lateral position than sitting [5]. The authors of the study posited that this finding may be due to extrinsic compression of the chest by abdominal contents in the lateral position.

Furthermore, studies have identified anatomic advantages in the sitting position that could explain greater success rates with LPs. Spinal ultrasound evaluation of infants in different positions showed that the sitting position increases interspinous process distance and subarachnoid space [6]. Specifically, sitting with maximal hip flexion provided the largest interspinous space based on bedside ultrasound evaluation of 51 infants in the neonatal intensive care unit [7]. In this study, the heart rate and oxygen saturation varied significantly with positioning of the infants but did not result in any apparent changes in clinical status.

In all previous studies, the infant sitting position was maintained manually. This study is the first in the literature to describe usage of a positioning device specifically designed for infant LPs (Smoltap^®^, Smoltap Inc., Providence, USA). The device, which has recently become commercially available, keeps infants in a stable sitting position and eliminates the need for holding them manually. The device can potentially improve procedural experience in a variety of clinical settings including the emergency department, inpatient floor unit, and neonatal intensive care unit. This device has recently become commercially available. However, published studies quantifying success rates associated with the device are still lacking. As we have adopted its use in our own IR practice, our study aim was to retrospectively evaluate the effectiveness and safety of the device for image-guided infant LPs.

## Materials and methods

This study was reviewed by the Institutional Review Board and was determined to be exempt.

We performed a retrospective study of infants who underwent image-guided LPs before and after the introduction of a positioning device. The study period spanned from May 2022 to April 2025, approximately 1.5 years before and after the introduction of the device in our IR department in October 2023. The device was used for infants who were stable on room air and with head-to-toe length of ≤ 57 cm per the instructions for use. The positioning device does not allow for placement of intubated patients. In addition, the positioning device does not allow for measurement of opening pressures, although none of the patients in our study required it. To allow for a valid comparison, the same inclusion criteria (on room air with head-to-toe length of ≤ 57 cm) were used for the group who underwent LPs prior to the introduction of the device.

Prior to the introduction of the positioning device, all infants were positioned in the conventional lateral flexed position. The infants were kept in position manually by a support staff or were sedated by a board-certified pediatric anesthesiologist. LPs were performed with either ultrasound or fluoroscopic guidance. Local anesthesia was administered in the form of topical lidocaine cream applied approximately 30 min prior to the procedure. A 22-gauge spinal needle was used for accessing the thecal sac. CSF samples were obtained and sent for laboratory studies as requested per the primary team.

**Fig. 1:**
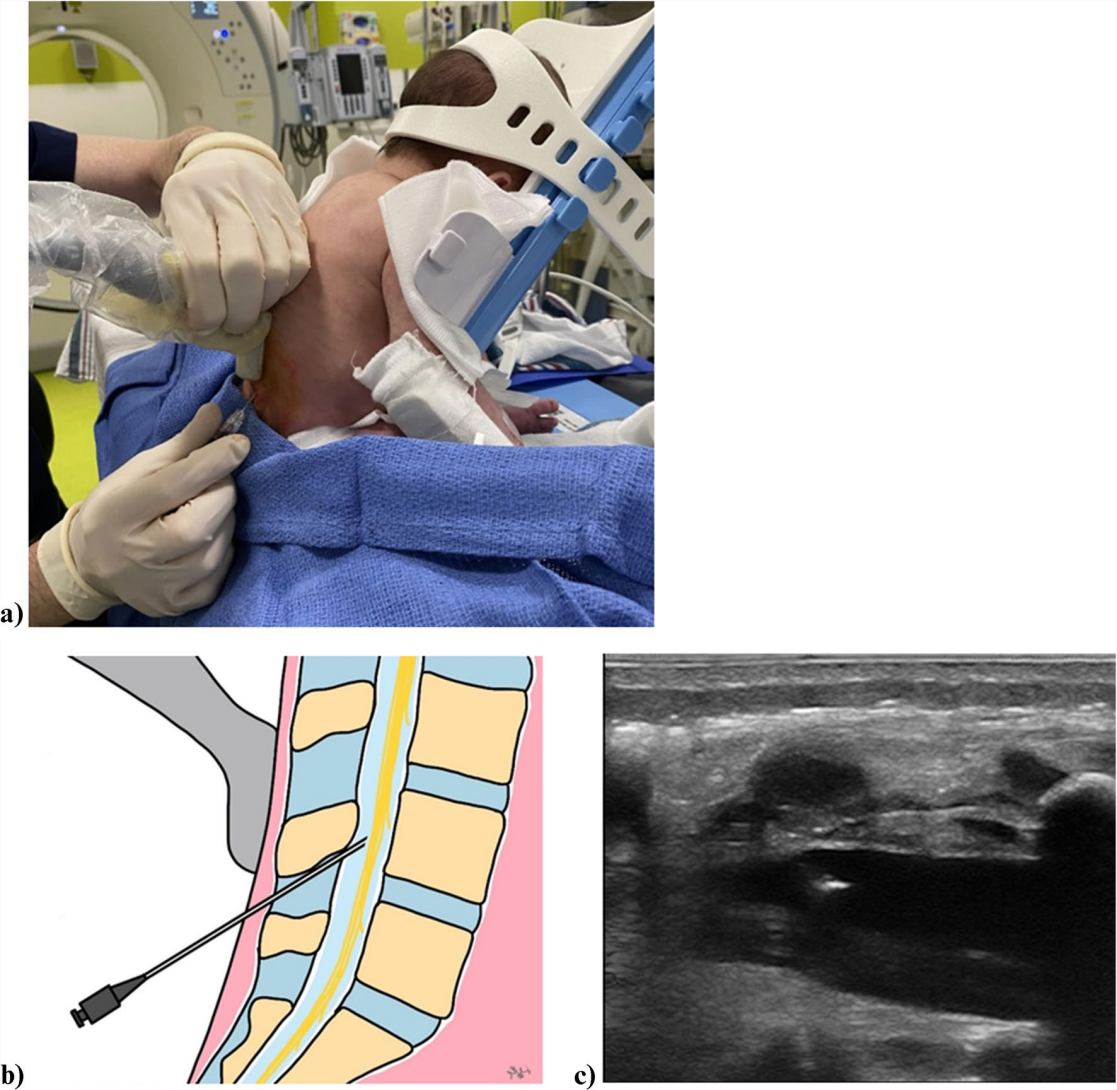
**a** Ultrasound-guided lumbar puncture using an infant positioning device. A 22-G spinal needle and a 14-Hz L-shaped “hockey stick” linear probe are used. **b** Pictorial illustration of the procedure. **c** Ultrasound image of the lower lumbar spine demonstrating the spinal needle tip within the thecal sac

Once the device became available in our department, infants were placed in the device in a sitting position according to its instructions for use [8]. The LPs were performed with real-time ultrasound guidance in sagittal approach (Fig. 1). The sitting position precluded the use of fluoroscopic guidance. The local anesthetic and needle type remained the same as above. In our practice, to ensure patient safety and procedural ergonomics, the device was secured with straps onto the procedure table prior to placing the infant, and once the patient was positioned in the device, the interventionalist sat on a low stool to comfortably perform LPs without excessively bending his/her back. In addition, sucrose solution was often used (> 50% of the time) as a soothing method where infants are encouraged to suckle on a gloved finger dipped in sucrose solution during the procedure. An interventional radiology nurse, present for every case to monitor the patient, sat facing the patient and provided the sucrose solution as needed.

The procedures were performed by three board-certified interventional radiologists. Patient and procedural data were collected by chart review. Statistical analysis was performed using a two-tailed *t* test and the chi-square test of independence (Excel, VassarStats). Significance level of < 0.05 was used.

## Results

We identified 42 infants who underwent 42 LPs with the positioning device (“device” group). Thirty-six infants underwent 37 LPs prior to the introduction of the positioning device (“no device” group). The demographic data between the two groups were similar (Table 1), including average age (17 days vs 20 days), weight (3.6 kg vs 3.6 kg), and head-to-toe length (51 cm vs 50 cm). The minimum/maximum weights and lengths of the infants in the “device” group were 2.4 kg/5.4 kg and 43 cm/56.6 cm, respectively. The infants were all under 3 months of age. The indication for nearly all infants was to assess for bacterial meningitis; other reasons included seizures and congenital syphilis.

**Table 1:** Demographics of the infants who underwent lumbar punctures with and without a positioning device

	Device (*n* = 42)	No device (*n* = 37)	*P*-value
Mean age, days (range)	17 (3–54)	20 (4–79)	0.36
Mean gestation, weeks (range)	39 (33–41)	38 (28–41)	0.49
Sex, males	26 (62%)	23 (62%)	0.98
Weight, kg (range)	3.6 (2.4–5.4)	3.6 (1.9–5.3)	0.70
Head-to-toe length, cm (range)	51.1 (43.0–56.6)	50.3 (44.0–57.0)	0.27

Of note, there were 19 infants who underwent LPs without the positioning device even after its introduction. These patients were not included in the main data analysis. Fourteen of them were intubated and one had a scrotal abscess, precluding them from placement into the positioning device. Two patients could not physically fit into the positioning device and therefore underwent conventional LPs with sedation. Their weight and head-to-toe length were 5 kg/52 cm and 5.9 kg/56 cm. For the remaining two patients, the reason for not using the positioning device was not documented in the chart.

The majority of LPs were preceded by failed bedside LPs without image guidance: 40 out of 42 (95%) in the “device” group and 32 out of 37 (86%) in the “no device” group (Table 2). The difference was not statistically significant (*P* = 0.17). While most bedside LPs resulted in a dry tap, 30% in the “device” group and 22% in the “no device” group resulted in a traumatic tap, either documented in the lab report as having erythrocyte count of ≥ 500/µL or in the procedure note as appearing frankly bloody. The difference was not statistically significant (*P* = 0.88). Spinal hematomas were seen on preprocedural ultrasound in 15 out of 40 cases (38%) in the “device” group. In the “no device” group, preprocedural ultrasound was available in 24 cases and of them 9 (38%) had spinal hematomas (*P* = 0.88).

**Table 2:** Procedural data of unsuccessful bedside lumbar punctures that infants underwent prior to repeat image-guided procedures with and without a positioning device

	Device (*n* = 42)	No device (*n* = 37)	*P*-value
Prior bedside LP	40 (95%)	32 (86%)	0.17
Prior traumatic tap	12 out of 40 (30%)	7 out of 32 (22%)	0.88
Spinal hematoma on US	15 out of 40 (38%)	9 out of 24 available US (38%)	0.88

In the “device” group, as described in the “Materials and methods” section, all LPs were performed with the patients awake and with ultrasound guidance only. In the “no device” group, 9 out of 37 LPs (24%) were performed with the patient awake and held in place manually, while the rest were sedated or under general anesthesia. Six out of the 9 awake procedures (67%) were performed with ultrasound guidance and the rest with fluoroscopy. Twelve out of the 28 sedated procedures (43%) were performed with ultrasound guidance and the rest with fluoroscopy.

CSF was obtained in 93% of cases in the “device” group and 84% in the “no device” group (Table 3). The difference was not statistically different (*P* = 0.21). The obtained CSF was adequate for microbial culture in 100% of cases in both groups, as well as for the FilmArray Meningitis/Encephalitis (FAME) panel, except for 2 cases (5%) in the “device” group due to low sample volume. The FAME panel is a polymerase chain reaction (PCR) test that simultaneously identifies 14 bacterial and viral pathogens [9]. The mean CSF volume obtained was 3.1 mL in the “device” group and 4.0 mL in the “no device” group. The difference was statistically significant (*P* = 0.03). At our institution, CSF volume of greater than 2 mL is usually enough for all necessary diagnostic tests for meningitis.

**Table 3:** Procedural outcomes of infant lumbar punctures performed with and without a positioning device

	Device (*n* = 42)	No device (*n* = 37)	*P*-value
CSF obtained	39 (93%)	31 (84%)	0.21
Diagnostic for microbial culture	39 out of 39 (100%), but 2 inadequate for FAME panel^1^	31 out of 31 (100%)	NA
Mean CSF volume, mL (range)	3.1 (0.5–6.0)	4.0 (0.5–6.0)	**0.03**
Traumatic tap:			
RBC ≥ 10,000 µL RBC ≥ 500 µL	10 out of 31 (26%) 28 out of 31 (72%)	5 out of 31 (16%) 13 out of 31 (42%)	0.42 **0.01**
Procedure duration, min (range)	14 (3–71)	16 (6–58)	0.65
Complications	0	0	NA

^1^FilmArray Meningitis/Encephalitis panel

Values in bold indicate statistical significance

With regard to traumatic or bloody taps, the definition varies in the literature [2, 10]. The criteria of ≥ 10,000 erythrocytes/µL and ≥ 500 erythrocytes/µL are frequently used. Using the former criterion, there were 10 traumatic taps (26%) in the “device” group and 5 (16%) in the “no device” group. Using the latter, more stringent criterion, the percentages were 72% and 42%, respectively. The difference was statistically significant under the latter criterion (*P* = 0.01) but not under the former (*P* = 0.42).

The mean procedure duration was 14 min in the “device” group and 16 min in the “no device” group, with the range of 3–71 min and 6–58 min, respectively. The difference was not statistically significant (*P* = 0.65). There were no incidences of post-procedural complications in either group.

The “no device” group was further broken down into awake and sedated subgroups (Table 4). There were no statistically significant differences observed between the “device” group and the awake “no device” subgroup in any of the procedural outcome metrics. Of note, the likelihood of obtaining CSF tended to be higher in the “device” group compared with the awake “no device” group (93% vs 78%, *P* = 0.17). Meanwhile, there were statistically significant differences between the “device” group and the sedated “no device” subgroup, specifically with regard to the mean CSF volume obtained (3.1 mL vs 4.3 mL, *P* = 0.002) and the percentage of traumatic taps defined by criterion of ≥ 500 erythrocytes/µL (72% vs 29%, *P* = 0.001).

**Table 4:** Subgroup analysis of infants who underwent lumbar punctures without a positioning device. The group is divided into those who did not receive intraprocedural sedation versus who did

	No device – AWAKE (*n* = 9)	*P*-value (compared to “device” group	No device – SEDATED (*n* = 28)	*P* value (compared to “device” group)
CSF obtained (%)	7 (78%)	0.17	24 (86%)	0.33
Mean CSF volume, mL (range)	2.5 (0.5–5)	0.34	4.3 (1.0–7.0)	**0.002**
Diagnostic for microbial culture	7 out of 7 (100%)	NA	24 (100%)^1^	NA
Traumatic tap:				
RBC ≥ 10,000 µL RBC ≥ 500 µL	3 out of 7 (42%) 6 out of 7 (86%)	0.29 0.44	2 out of 24 (8%) 7 out of 24 (29%)	0.09 **0.001**
Procedure duration, min (range)	21 (7–40)	0.20	13.7 (6–58)	0.78
Complications	0	NA	0	NA

Values in bold indicate statistical significance

## Discussion

Our experience with the infant positioning device supports its feasibility and safety for performing image-guided LPs. LPs performed with the device had 93% success rate of obtaining adequate CSF for microbial analysis with 0% complication rates. The procedural outcomes of LPs before and after the implementation of the device were largely similar, including the procedural duration. There was no statistically significant difference in the proportions of traumatic taps when defined as CSF containing ≥ 10,000 erythrocytes/µL (26% and 16%, *P* = 0.42). When defined as ≥ 500 erythrocytes/µL, however, the rates of traumatic taps were significantly different (72% and 42%, *P* = 0.01).

This difference is most likely due to inclusion of sedated patients in the “no device” group. A subgroup analysis within the “no device” group showed that infants who received intraprocedural sedation had much lower incidence of traumatic taps than those who did not (29% versus 86%, *P* = 0.008). When only the latter subgroup was compared to the “device” group, in which no patients were sedated, there was no statistically significant difference in the incidence of traumatic taps (86% versus 72%, *P* = 0.20). It makes intuitive sense that sedation, by minimizing patient movement, is likelier to decrease the chance of injuring the vertebral plexus during needle advancement and therefore the rate of traumatic taps. The device, on the other hand, cannot eliminate patient crying or movement. The lack of sedation in the “device” group also likely contributed to the lower CSF volume that was able to be obtained compared to the sedated “no device” group (3.1 mL versus 4.3 mL, *P* = 0.002).

There is paucity of data on the incidence of traumatic taps in image-guided infant LPs. A large study involving 1,240 neonatal (≤ 28 days old) non-image-guided LPs showed that the incidences of bloody taps were 42.9% under the criterion of ≥ 500 erythrocytes/µL, and 16.6% under the criterion of ≥ 10,000 erythrocytes/µL [11]. The study also found that if the first tap was bloody, the odds ratio of the second being also bloody was 5.8. Another study also suggested that blood can leak into the CSF after traumatic LPs and can take up to 2 weeks to clear [12]. Given that the image-guided LPs in our study almost always followed multiple failed bedside attempts 1–3 days prior, it is reasonable to expect higher incidence of traumatic taps in our study than the above reported incidences.

The subgroup analysis suggests a trend in which the procedural outcomes were more favorable in the “device” group compared to the awake “no device” subgroup. As discussed, the incidence of traumatic taps tended to be lower in the “device” group (72% vs 86%) although not statistically significant. In addition, the mean procedure duration was shorter (14 min vs 21 min, *P* = 0.20). We noted a trend toward shorter procedure duration with the device over time, with a mean of 24 min in the first 6 months of device use and 11 min in the rest of the study period, likely due to operators becoming more experienced with the device over time. Prospective study with greater sample size will help to confirm whether the device is in fact associated with significantly better procedural outcomes.

There are additional qualitative advantages associated with the device. It is not always safe or practical to administer sedation in every infant that needs an image-guided LP. Prior to the introduction of the device, over 75% of the infant LPs were performed with anesthesia in our practice. After the introduction, we were able to perform LPs without anesthesia on most infants unless they did not fit in the device or if they were already intubated. Given limited anesthesia resources, this helped to free up anesthesia time for other cases in our practice. Also, fluoroscopy is not used with the device; therefore, no ionizing radiation is delivered to the patient. Additionally, another benefit is reduced stress for support staff as the device eliminates the need for manually holding down infants for the entire duration of the LP and CSF collection.

In our experience, we found that the body weight can be a useful predictor for whether an infant will fit into the device. The instructions for use of the device currently provide only the head-to-toe length limit of < 57 cm for infants but not a weight limit. The maximum weight of the infant in our cohort to fit into the device was 5.4 kg. There were two infants under the length limit but weighing 5 kg and 5.9 kg whose shoulders could not fit into the device. We suggest using weight has an additional metric in determining whether a patient is an appropriate candidate for the device, with 5–5.5 kg as a reasonable weight limit.

The main limitation of the study is the lack of a simultaneous head-to-head comparison between the “device” and “no device” groups. The study used a historical control group that underwent LPs prior to the device being available. The control group was somewhat heterogeneous, reflecting the practice pattern at the time. The majority (> 75%) of infants prior to device implementation underwent LPs with sedation. The awake “no device” subgroup, which was a better matched control to the “device” group, was therefore small (*n* = 9). In addition, there were other heterogeneities in the study sample that could introduce bias, such as use of fluoroscopy and ultrasound guidance in the “no device” group as well as changes in the interventional physician staff over time. Ideally, we need a prospective randomized study of infants undergoing awake ultrasound-guided LPs with and without the device to overcome these limitations.

However, the fact that the proportion of sedated versus awake LPs reversed over time in our institution also sheds light unto the impact the device had on our practice. Prior to the implementation of the device, sedation was regularly utilized to increase the success rate of LPs. However, as the interventionalists became comfortable with the use of the device and felt that the device was adequately securing the infants for obtaining CSF, performing awake LPs with the device became the preferred method. Given its effectiveness and relative ease of implementation, the device can be relevant to many pediatric IR practices.

## Conclusion

The use of an infant positioning device to perform ultrasound-guided LPs is feasible and safe. There are several benefits to using the device. Infants do not need to be sedated or held manually to maintain stable positioning. The device keeps the infants in a sitting position, which has been shown to be anatomically advantageous for performing LPs compared to the conventional lateral flexed position. A possible downside of the device may be higher rates of traumatic taps with the device than without. However, this finding can be attributed to inclusion of sedated patients in the control group, as our subgroup analysis suggests. A controlled prospective trial is needed in the future to better investigate this potential association. It is also important to note that the device is not appropriate for infants who are intubated or too large for the device (> 57 cm in head-to-toe length and, in our experience, over 5–5.5 kg in weight).

## Data Availability

No datasets were generated or analysed during the current study.
